# Early herpes and TTV DNAemia in septic shock patients: a pilot study

**DOI:** 10.1186/s40635-019-0256-z

**Published:** 2019-05-18

**Authors:** François Mallet, Magali Perret, Trang Tran, Boris Meunier, Audrey Guichard, Olivier Tabone, Marine Mommert, Karen Brengel-Pesce, Fabienne Venet, Alexandre Pachot, Guillaume Monneret, Frederic Reynier, Christophe Védrine, Philippe Leissner, Virginie Moucadel, Alain Lepape, Julien Textoris, MIPrea group, André Boibieux, Julien Davidson, Laure Fayolle-Pivot, Julie Gatel, Charline Genin, Arnaud Gregoire, Alain Lepape, Anne-Claire Lukaszewicz, Guillaume Marcotte, Marie Matray, Delphine Maucort-Boulch, Nathalie Panel, Thomas Rimmelé, Hélène Vallin, Fabienne Venet, Sophie Blein, Karen Brengel-Pesce, Elisabeth Cerrato, Valérie Cheynet, Emmanuelle Gallet-Gorius, Audrey Guichard, Camille Jourdan, Natacha Koenig, François Mallet, Boris Meunier, Virginie Moucadel, Marine Mommert, Guy Oriol, Alexandre Pachot, Claire Schrevel, Olivier Tabone, Julien Textoris, Javier Yugueros-Marcos, Jérémie Becker, Frédéric Bequet, Yacine Bounab, Florian Brajon, Bertrand Canard, Muriel Collus, Nathalie Garcon, Irène Gorse, Cyril Guyard, Fabien Lavocat, Philippe Leissner, Karen Louis, Maxime Mistretta, Jeanne Moriniere, Yoann Mouscaz, Laura Noailles, Magali Perret, Frédéric Reynier, Cindy Riffaud, Mary-Luz Rol, Nicolas Sapay, Trang Tran, Christophe Vedrine, Christophe Carre, Pierre Cortez, Aymeric De Monfort, Karine Florin, Laurent Fraisse, Isabelle Fugier, Maïna L’Azou, Sandrine Payrard, Annick Peleraux, Laurence Quemeneur, Andrew Griffiths, Stephanie Toetsch, Teri Ashton, Peter J. Gough, Scott B. Berger, David Gardiner, Iain Gillespie, Aidan Macnamara, Aparna Raychaudhuri, Rob Smylie, Lionel Tan, Craig Tipple, REALISM group, Guillaume Marcotte, Christian Guillaume, Romain Hernu, Sylvie De La Salle, Marie Simon, Thomas Baudry, Elisabeth Cerrato, Emmanuelle Gallet-Gorius, Audrey Larue-Triolet, Christine Alberti-Segui, Nathalie Panel, Marion Provent, Mathieu Page, Anne Portier

**Affiliations:** 1 EA 7426 Pathophysiology of Injury-induced Immunosuppression, University of Lyon1-Hospices Civils de Lyon-bioMérieux, Hôpital Edouard Herriot, 5 Place d’Arsonval, 69437 Lyon Cedex 3, France; 2 Joint research unit, Hospice Civils de Lyon, bioMérieux, Centre Hospitalier Lyon Sud, 165 Chemin du Grand Revoyet, 69310 Pierre-Benite, France; 3 BIOASTER Technology Research Institute, Lyon, France; 4 Soladis, Lyon, France; 5 Hospices Civils de Lyon, Immunology Laboratory, Groupement Hospitalier Edouard Herriot, Lyon, France; 6 Intensive Care Unit, Centre Hospitalier Lyon Sud, Hospices Civils de Lyon, Pierre Bénite, France; 7 Emerging Pathogens Laboratory, Epidemiology and International Health, International Center for Infectiology Research (CIRI), Lyon, France; 8 Hospices Civils de Lyon, bioMérieux Joint Research Unit, Groupement Hospitalier Edouard Herriot, Lyon, France; 9 Hospices Civils de Lyon, Department of Anaesthesiology and Critical Care Medicine, Groupement Hospitalier Edouard Herriot, Université Claude Bernard Lyon 1, Lyon, France

**Keywords:** Sepsis, Immunosuppression, Mortality, Biomarker, Herpes viruses, EBV, HSV1, CMV, HHV-6, TTV

## Abstract

**Background:**

Septic shock patients exhibit an increased incidence of viral reactivation. Precise timing of such reactivation—as an early marker of immune suppression, or as a consequence of the later—is not known precisely. Here, using a fully designed nucleic acid extraction automated procedure together with tailored commercial PCR kits, we focused on the description of early reactivation within the first week of ICU admission of several herpes viruses and Torque Teno virus (TTV) in 98 septic shock patients.

**Results:**

Most of septic shock patients had at least one viremia event during the first week (88%). TTV and herpesviruses were detected in 56% and 53% of septic shock patient, respectively. The two most frequent herpesviruses detected within the first week were EBV (35%) and HSV1 (26%). Different kinetic were observed among herpesviruses, faster for EBV and HSV1 than for CMV and HHV6. Although no association was found between herpes viremia and secondary infections, patients with herpesviridae-related viremia were more severe, e.g., higher SOFA scores and plasma lactate levels. While reactivating only 1 virus was not associated with mortality, patients with multiple viremia events had higher ICU mortality. Surprisingly, EBV + TTV early reactivation seemed associated with a lower D28 mortality. No clear association was observed between viremia and immune biomarkers.

**Conclusion:**

Applying a semi-automated process of viral DNAemia determination to this cohort of 98 patients with septic shock, we observed that the number of patients with positive viremia increased during the first week in the ICU. Of note, there was no improvement in predicting the outcome when using viremia status. Nevertheless, this pilot study, introducing standardized procedures from extraction to detection, provides the basis for future standardized diagnostic criteria. A prospective longitudinal clinical study using these procedures will enable determination of whether such viremia is due to a lack of a latent virus control by the immune system or a true clinical viral infection.

**Electronic supplementary material:**

The online version of this article (10.1186/s40635-019-0256-z) contains supplementary material, which is available to authorized users.

## Take home message

Herpes viruses and TTV viremia increase as early as the first week at the ICU. Multiple viremia appeared associated with mortality, while EBV + TTV co-infections appeared to contribute to an apparent protective effect regarding mortality.

## Background

Septic shock represents the most severe state of a dysregulated host response to infection. In the last 10 years, there has been an improved understanding of sepsis pathophysiology and clinical management [1], and in particular, a central role for immunosuppression in sepsis [2]. Several studies demonstrated a significant association between immune alterations and an increased incidence of secondary infections (reviewed in [3]). Among these, septic shock patients exhibit an increased incidence of viral reactivation. Whether such viremia is a lack of latent virus control or true clinical viral infection is still a matter of debate.

Torque teno virus (TTV), a non-enveloped single-strand DNA virus of the genus Anellovirus, was similarly detected in adult [4] and pediatric septic patients [5], suggesting it might also provide information on the immune status of these patients. Indeed, qualitative and quantitative altered states of the plasma virome were observed in tacrolimus [6] or HIV-1 [7]-induced immunosuppression, highlighting the correlation between the Anellovirus viral load and the extent of immunosuppression. Moreover, herpes viruses have been shown to be reactivated in adult [4, 8, 9] and, to a differing extent, in pediatric sepsis patients [5], as well as in ostensibly immunocompetent critically ill patients [10, 11]. Interestingly, such viral reactivation was also associated with an increased incidence of fungal infections [4]. Furthermore, it remains unknown whether prophylactic or pre-emptive initiation of antiviral drugs, such as acyclovir or ganciclovir, in septic patients should occur: the recent failure to demonstrate efficacy in studies of such patients may be related to a lack of stratification [12–15]. Monitoring of a panel of herpes viruses which persist in the host might provide information about the type or degree of immunosuppression and guide clinical management.

Here, we focus on the description of early reactivation (within the first week of ICU admission) of several herpes viruses (CMV, EBV, HSV1, and HHV6) and TTV in septic shock patients. Using a fully designed nucleic acid extraction automated procedure together with tailored commercial kits, we defined the limit of detection (LOD) criteria enabling qualitatively significant viral reactivations to be addressed. Secondary objectives explored the association of viral DNAemia with (i) clinical outcomes such as mortality and health-care associated infections (HAI), (ii) molecular markers, e.g., CD74 or IL10 [16], CX3CR1 [17], and IL-1β [18], and (iii) whether early viral DNAemia combined with immune molecular markers better predict HAI and mortality.

## Methods

### Biological samples

#### Viral standards

Virus strain-reference panel (Additional file 1: Table S1A) consisting of CMV AD169 (Cytomegalovirus), EBV B-95 (Epstein-Barr virus), HSV1 95 (herpes simplex virus type 1) (Qnostics Molecular “Q” panels), and HHV6 Z29 (Human Herpes Virus 6) (ZeptoMetrix NATtrol-Molecular controls) were used to define the limit of detection (LOD) for all viruses in the semi-automated process. All of these virus strains have also been used as external extraction positive controls, using the last positive dilution (LOD, see below).

#### Patient cohort and healthy controls

We retrospectively selected patients from a prospective, multicentre, non-interventional study conducted in six ICUs in Lyon, France (Additional file 1: Figure S1). The study was approved by our institutional ethical review board (Comité d’Ethique des Centres d’investigation Clinique de l’Inter-Région Rhône-Alpes Auvergne–IRB 5044) and consent for ancillary study was obtained a posteriori. The protocol of this ancillary retrospective study was submitted to the French CCTIRS and CNIL committees and approved on 22/04/2016 and 30/09/2016, respectively. Further, informed consent was received from patients for inclusion in this specific study. The cohort is described in details elsewhere [16]. For the current study, we selected all the patients from the septic shock group for which all D1, D3/4, and D5/7 plasma samples were available.

Citrated pouches or heparinized blood tubes from healthy individuals were obtained from EFS (Etablissement Français du Sang) and used immediately. According to EFS standardized procedures for blood donation and to provisions of the articles R.1243–49 and following procedures of the French Public Health Code, a written statement confirming non-opposition to the use of their blood donation for research purposes was obtained from healthy volunteers. The blood donors’ personal data were anonymized before transfer to our research lab. We obtained the approval notice of the Local Ethical Committee (Comité de Protection des Personnes Sud-Est II, Bâtiment Pinel, 59 Boulevard Pinel, 69,500 Bron) and the acceptance of the Ministère de la Recherche (declaration DC-2008-64) for handling and conservation of these samples.

### Sample treatment/nucleic acid extraction

The overall procedure is depicted in Additional file 1: Figure S2. The extractions of viral DNA were carried-out using the Maxwell® HT Viral TNA chemistry (Promega) consisting of paramagnetic silica particles and the liquid handling robot Freedom EVO® (TECAN). A custom automation procedure was developed at BIOASTER (script extract_virale _promega_V5_2). Briefly, 10 μl of internal control (IC2) provided in R-GENE® kit was spiked into 200 μL of each plasma sample (IC2 sample), positive control, or negative control (IC2W0). The samples were lysed in the presence of proteinase K at 65 °C for 10 min, then the nucleic acids were captured by the paramagnetic silica particles, washed, and eluted in 65 μl of nuclease-free water. After applying a batching procedure to minimize bias), all the samples were extracted 4 times (3 plates of 72 samples and 1 plate of 90).

### Viral DNAemia determination

#### LOD determination

Using virus-free plasma loaded with a predetermined quantity of target virus reference strains (see above), LOD was determined as the lowest quantity (genome copy) that can be distinguished from the absence of detection (a blank value) with a specified confidence level (generally 99%). Firstly to determine the LOD, the control plasma was diluted in series from 10^−1^ to 10^−4^, then each diluted sample was extracted independently 3 times and amplified according to the qPCR protocol described below, to determine an approximate limit of detection. Then in a second step, the last dilution giving a positive signal was further diluted to 1/2, and 1/4 and each diluted sample was extracted 20 times and amplified. We used the most diluted positive replicates with 100% of detection as the LOD. The overall process as well as the last dilution and the amplification of technical control samples from unmatched healthy volunteers are depicted in Additional file 1: Figure S3.

#### Standardized quantitative real-time PCR

DNA viruses real-time PCR reactions were performed on the StepOnePlus™ Real-Time PCR System (ThermoFisher SCIENTIFIC) using R-GENE® assay kits for CMV, EBV, HSV1, HHV6, and TTV (bioMérieux SA) (Additional file 1: Table S1B). Virus R-GENE® kits consist of virus-specific primer pairs for amplification, and the real-time detection of the amplified product is achieved using a TaqMan probe. The amplification master mix contains the amplification primers, the dNTPs, the amplification buffer, the Taq Polymerase, and the viral probe as well as the primers and probes specific for the internal control which must also be subjected to the entire procedure of extraction. Internal controls (IC2 and IC2W0) are provided to evaluate the extraction efficiency and to detect the presence of possible inhibitors. A range of four quantification standards (QS), one sensitivity control (SC) and one negative control (water), are supplied to control quantitation efficiency and the absence of contamination. The overall process is depicted in Additional file 1: Figure S2. Briefly, all sample DNAs, randomly batched in four plates, were simultaneously amplified with quantification standards, sensitivity control, and negative control. qPCR was performed according to the manufacturer’s instructions in a 25-μl volume containing 15 μl of the amplification premix and 10 μl of standard or sample DNA. The PCR protocol consisted of 45 cycles of 10 s denaturation at 95 °C, 40 s annealing and extension at 60 °C. Fluorescence data were acquired on each cycle at the end of the annealing step. After amplification, the results were validated with extraction, inhibition, positive, and negative controls. According to the supplier, IC2W0 should not give any signal (CT, crossing threshold) at 530 nm but should give a signal less than or equal to 32 cycles at 560 nm. The comparison of CT values for both IC2W0 and IC2 sample allowed to evaluate the efficiency of the extraction and to detect the presence of possible inhibitors. If (Ct [IC2 sample] ≤ Ct [IC2W0] + 3), the sample was considered as not inhibited. For the quantification, the CT value (at 530 nm) for QS3 and the slope obtained from the standard curve using QS1–4 should be within the values provided by the supplier. Finally, using the standard curve, samples CT are converted into copies per microliter of viral DNA in the PCR reaction tube, then in copies per milliliter of viral DNA in the plasma.

### Cytokines and immune markers

We benefited from the previous assessment at the molecular level of various cytokines and immune markers (CD74, CX3CR1, IL10, IL-1β) performed on the MIPrea cohort as described elsewhere [16, 17]. Briefly, biomarkers, quantified by RT-PCR, consisted of CD74 ratio of D3/D1 (> 1.238 = increased incidence of HAI) and IL10 measured at D3 (> 0.039 = increased incidence of HAI) [16], CX3CR1 measured at D3 (> 0.253 = increased incidence of mortality at D28) [17], and IL1b measured at D3 (increase at D2-D4 = increase incidence of HAI) used as a continuous variable [19].

### Statistical analysis

Within the first week after ICU admission, for each virus and for each patient, early viremia has been defined as positive if at least one of the patient’s samples (day 1, day 3, or day 6) was positive. The positivity is set by a level of a virus copy number per microliter above a pre-defined threshold (LOD).

Association between viremia event and clinical outcomes (survival or health-care associated bacterial infection as previously described [16]) was assessed using *χ2* test or Fisher’s exact test where appropriate. The same tests were used to investigate the association between viremia event and dichotomized molecular markers according to previously described thresholds [16, 17]. Analyses were conducted with R version 3.4.4 software and statistical significance was defined by an alpha-risk *p* < 0.05.

## Results

### Cohort description

To monitor early viremia in septic shock patients, we screened a previously described cohort of 749 ICU patients with systemic inflammatory response [16] and included 98 patients out of the 262 septic shock patients from the initial cohort (Additional file 1: Figure S1). This subgroup was similar in terms of severity at admission but exhibited a non-significant higher incidence of secondary infections, and a more prolonged ICU stay (14 days [9–21] vs 7 days [4–15]; *p* value = 0.001).

Most patients had at least one viremia event during the first week (*n* = 86, 88%), 52 (53%) patients had at least 1 herpesviridae-related viremia during the first week (Table 1). Patients with herpesviridae-related viremia were more severely unwell (higher SOFA score over the first week, increased need for hemofiltration, higher plasma lactate level). This higher severity is illustrated by a lower expression of CX3CR1 (0.20 [0.07–0.24] vs 0.27 [0.13–0.38]; *p* < 0.01), a prognostic marker we recently confirmed in ICU patients [17]. Patients with at least one herpesviridae-related viremia had a lower production of IL-1β (1.15 [0.87–1.82] vs 1.64 [1.11–2.39]; *p* < 0.01), a cytokine suggestive of trained immunity [18]. A higher proportion of women had a viremic episode (23 (44%) vs 10 (22%), *p* = 0.03). Of note, a lower leukocyte count at admission, or an increased use of hydrocortisone, was not observed in the viremic patients. In septic shock patients that were still in ICU at day 7, viremia with at least 1 herpesviridae during the first week was not associated with a worse outcome (measured either through organ support duration, length of stay, or mortality).

**Table 1:** Septic shock patient characteristics at admission and molecular markers and outcomes according to viral DNAemia during the first week in ICU (excluding TTV)

	Viremia not present (*n* = 46)	At least one herpes viremia event (*n* = 52)	Whole cohort (*n* = 98)	*p* value
Demographics				
Gender (female)	10 (21.7)	23 (44.2)	33 (33.7)	0.03*
Age (years)	70 [61–75]	69 [57–78]	70 [59–77]	0.86
Admission data				
SAPS II	62 [49–72]	64 [56–74]	63 [52–72]	0.18
SOFA score day 1	11 [8–13]	12 [10–14]	12 [9–14]	0.01*
SOFA score day 3	8 [7–12]	11 [8–14]	9 [7–13]	0.02*
SOFA score day 6	5 [4–8]	7 [5–11]	6 [4–10]	0.03*
Hemofiltration	7 (15)	20 (38)	27 (28)	0.02*
Charlson score	2 [1–4]	1 [0–2]	2 [1–3]	0.04*
Primary site of infection				0.19
Pulmonary	27 (59)	26 (50)	53 (54)	
Abdominal	12 (26)	10 (19)	22 (22)	
Other	7 (15)	16 (31)	23 (23)	
Type of primary infection				0.14
Community acquired	27 (59)	38 (73)	65 (66)	
Hospital acquired	19 (41)	14 (27)	33 (34)	
Hydrocortisone	27 (60)	40 (78)	67 (70)	0.08
Chemistry and hematology at admission				
Plasma lactate level (mM)	2.3 [1.6–3.2]	2.9 [2.2–4.9]	2.6 [1.8–4.0]	0.02*
Neutrophils (G/L)	8.4 [6.0–13.1]	12.0 [7.3–15.6]	9.9 [6.5–14.6]	0.17
Lymphocytes (G/L)	0.6 [0.4–1.0]	0.8 [0.5–1.1]	0.7 [0.4–1.1]	0.33
Monocytes (G/L)	0.4 [0.2–0.8]	0.3 [0.1–0.7]	0.4 [0.1–0.8]	0.29
Molecular markers				
CD74 ratio (D3/D1; CNRQ)	1.2 [0.8–1.6]	1.3 [0.7–1.7]	1.2 [0.7–1.7]	0.77
CX3CR1 day 3 (CNRQ)	0.27 [0.15–0.52]	0.17 [0.08–0.29]	0.21 [0.10–0.38]	< 0.01*
IL10 day 3 (NRQ)	0.04 [0.03,0.06]	0.03 [0.02,0.06]]	0.04 [0.02,0.06]	0.22
IL1β day 3 (CNRQ)	1.64 [1.11–2.39]	1.15 [0.87–1.82]	1.34 [1.05–2.14]	< 0.01*
Outcomes				
Vasopressors duration (days)	2.9 [1.8–6.0]	3.4 [2.1–5.7]	3.1 [1.9–6.0]	0.66
Hemofiltration duration (days)	4 [3–10]	4 [2–6]	4 [2–7]	0.55
Mechanical ventilation (days)	9 [7–17]	11 [8–16]	11 [7–17]	0.45
ICU length of stay (days)	13 [9–24]	16 [10–20]	14 [9–21]	0.21
Hospital length of stay (days)	28 [16–53]	33 [19–59]	30 [19–54]	0.44
ICU mortality	11 (24)	12 (23)	23 (23)	1.00
Survival D28	35 (76)	42 (81)	77 (79)	0.75
At least one IAI	11 (24)	14 (27)	25 (26)	0.91

Categorical variables are expressed as *n* (%) and continuous variables as median [Q1–Q3]. Comparisons between “at least one viral event” and “no viral DNAemia” were performed with chi-squared test for qualitative variables and Mann-Whitney or *t* tests for quantitative variables, as appropriate. Values labeled with * indicate significance at *p* < 0.05. *IAI * ICU aquired infection, *ICU* intensive care unit, *SAPSII* simplified acute physiology score, *SOFA* sequential organ failure assessment, *(C)NRQ* PCR (calibrated) normalized relative quantities

### Viremia in septic shock patients during the first week in ICU

**Fig. 1:**
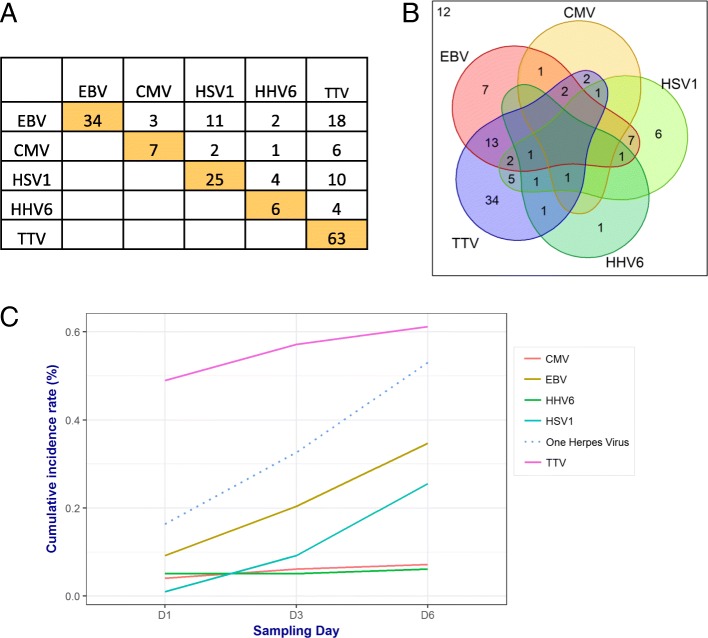
DNAemia during the first week following admission. **a** Number of patients presenting single- or multiple-positive herpes and/or TTV viral DNAemia during the first 6 days following admission in the ICU. Occurrence of each type of herpes virus (HV) or TTV is depicted in the orange boxes. Co-occurrence of HV with other HV or TTV is reported in white boxes. **b** Venn diagram illustrating single versus multiple viral reactivations; 12 patients presented with no viral event. **c** Cumulative incidence of individual or collective HV and TTV DNAemia at days 1, 3, and 6 following admission in the ICU

As pleiotropic herpesviruses may develop lifelong latency in their human host with reactivation during periods of immunosuppression whatever its origin [20–22], we first considered herpesviridae-related viremia as a whole, i.e., herpesviruses were grouped as a surrogate of immune failure. Among the 52 (53%) patients who had at least one herpesviridae-related viremia during the first week, 35 (36%), 14 (14%) and 3 (3%) patients respectively presented with 1, 2, or 3 different herpes viruses (co-) reactivation, while none were detectable in 19 (100%) healthy volunteers. The two most frequently detected viruses were EBV (*n* = 34, 35%) and HSV1 (*n* = 25, 26%) (Fig. 1a). CMV (*n* = 7, 7%) and HHV6 (*n* = 6, 6%) detection was considerably lower. EBV and HSV1 were the most frequently observed herpesvirus co-infection (*n* = 11, 11%). There were also two occurrences of triple EBV, HSV1, and HHV6 co-infection detected (Fig. 1b).

TTV was detected in 55 (56%) septic shock patients, almost three times more than in healthy controls (20%). Thirty-four (35%) patients expressed TTV only, while TTV was co-detected with 1, 2, or 3 herpes viruses in 21 (21%), 6 (6%), and 2 (2%) patients, respectively. The two most frequent herpesviruses co-detected with TTV were EBV (*n* = 27, 28%) and HSV1 (*n* = 13, 13%). Overall, the detection of only a single herpes virus or TTV was the most frequent occurrence (49%).

The cumulative incidence of viral DNAemia (Fig. 1c) was below 9% for each type of herpesvirus and 49% for TTV at ICU admission. The cumulative occurrence of herpesvirus DNAemia increased from 18 to 53% during the first week, while there was a smaller increase in TTV DNAemia during this period (49 to 61%).

### Association of viremia with clinical outcomes

Association between viremic event and clinical endpoints (survival, secondary infections) was investigated. Due to their low occurrence, CMV and HHV6 viremia were not considered. No statistical association was found between EBV, TTV, or HSV1, and D28 survival status (Additional file 1: Table S2). Similarly, no association was found between herpesvirus viremia and secondary infections. Although a small association was observed for EBV, this should be confirmed in a larger patient cohort (*p* = 0.076).

Patients with multiple viremic events had higher ICU mortality (29%) compared to those with a viremia from a single virus (14%), but mortality was similar when compared to patients with no viremia (25%) (*χ2* test, *p* = 0.39). Meanwhile, patients with multiple viremic events had similar HAI episodes (24%) compared to those with either a viremia from a single virus (29%) or no viremia (25%) (*χ2* test, *p* = 0.95).

As we previously described, CD74/IL10 and CX3CR1/IL1b are surrogate biomarkers of HAI and survival respectively [16, 17, 19]. Patients that reactivated at least one herpesvirus had a significantly lower CX3CR1 and IL1b (which are molecular markers associated with survival) but similar CD74 and IL10 levels (usually associated with HAI). The association between molecular biomarkers, dichotomized according to previously described thresholds (cf materials and methods), and viremia was also evaluated for EBV, TTV, and HSV1. EBV reactivation seemed to drive this trend (Additional file 1 Table S2).

## Discussion

In order to properly evaluate the use of virus reactivation as a prognostic (not an etiologic) marker during the very first week after ICU admission, we proposed an easily implementable fully designed automated procedure including extraction and amplification controls, together with a careful LOD determination. In this study, we thus provide a detailed description of TTV and herpesviridae-related viremia during the first week of ICU admission for septic shock; at least one virus was detected at early timepoints in more than 80% of these patients. Our study adds to the literature evaluating viral reactivation in patients with sepsis summarized in Table 2.

**Table 2:** Cumulative percentages of reactivation of various herpes viruses and TTV in septic patients during ICU stay

Reference	This study	Walton AH et al. 2014 [4]	Ong DSY et al. 2017 [8]	Brenner T et al. 2012 [9]	Davila S et al. 2017 [5]
Description of cohort	Septic shock patients, in first week of adult ICU stay (*n* = 98) (%)	Septic adult patients (*n* = 560) (%)	Septic shock patients (*n* = 329) (%)	Septic shock patients (*n* = 60) (%)	Septic pediatric patients (*n* = 73) (%)
EBV	35	53	48		11
HSV1	26	14	26	52	4
CMV	7	24	18	27	5
HHV6	6	10	24		8
TTV	64	77			89

EBV was detected in 35% of patients during the first week in ICU in our study. This detection rate is lower than previously observed in septic adult patients during their whole ICU stay [4, 8], but considerably higher than in pediatric patients, who may not yet have undergone EBV primary infection [5]. Large variations in EBV reactivation have been described in critically ill patients in the ICU including immunocompetent patients, ranging from 23 (7 to 28 days) [10] to 68% (median length for positivity of 7.5 days) [11]. HSV1 was detected in 26% of our patients, which is within the range of what has been observed in septic adult patients [4, 8, 9], but higher than observed in an undifferentiated adult ICU population (12%) [23] or septic pediatric patients (4%) [5]. CMV was detected in 7% of our patients during the first week at ICU, which is lower than observed in septic adult patients during the whole ICU stay [4, 8, 9], but in the same range as pediatric patients [5]. Similarly, HHV6 was detected in 6% of our patients, which is again lower than generally observed in septic adult patients during the whole ICU stay [4, 8], but in the same range than observed in pediatric patients 8% [5]. TTV was detected in 64% of our patients, slightly lower than observed in septic adult [4] and pediatric patients [5].

Although EBV and TTV were the viruses most frequently co-detected (18%), those viruses were predominantly reactivated separately (EBV 16/34, 47% and TTV 45/63, 71%); this seems to exclude any helper effect of EBV infection in TTV replication as previously described [24]. Moreover, EBV was generally reactivated in the absence of other herpes viruses (20/34, 59%). In contrast, other herpes viruses were mainly reactivated with other viruses, i.e., CMV (5/7; 71%), HHV-6 (4/6; 67%), and HSV1 (14/25, 56%). Among the few CMV reactivations, only 2 out of 7 (29%) were HSV1 positive and 1 (14%) was HHV6 positive; this is less than the 66% of CMV reactivators who were also HSV positive [25] and 16 to 23% of CMV reactivators who were also HHV6 positive [26] observed in other studies.

Of note, the drastically different kinetics observed for the herpes viruses, with EBV and HSV1 differing from CMV and HHV6, may reflect differences in the extent and duration of immunosuppression and therefore could be linked with clinical outcomes.

Overall, with the exception of EBV-TTV, patients with multiple viremic events had the highest ICU mortality (29%), a finding that has been previously observed [8]. No association between EBV detection in the first week of admission and mortality was found in our study. EBV viremia has been associated with mortality in patients with long ICU stays [11], but this was not confirmed elsewhere [4, 8]. We observed a trend between EBV detection in the first week of admission and HAI. Of note, an association between EBV in plasma and increased fungal infections has been suggested [4]. We capitalized on previous work showing an association between immune markers and mortality [17] or HAI [16]. While no association between EBV DNAemia and CD74 (a proxy of HAI) was observed in our study, there was a significant association with CX3CR1 (a proxy of mortality) was evidenced.

In our study, the early co-reactivation of EBV-TTV seemed to be associated with a lower D28 mortality (92% of survivors versus 76% for non-EBV-TTV reactivation), and EBV-HSV1 followed the same trend (86% versus 78%). Non-clinical studies have demonstrated that mice with low-level EBV-like infection have improved survival in bacterial sepsis [27]. Moreover, lower 90-day mortality has been observed in patients exhibiting low EBV viral load (< 4000 cp/ml) in the blood (but not plasma) [4]. Such an effect may be due to EBV encoded proteins or small RNAs (dUTPase, GP350, EBERs) that can directly sense toll-like receptors [28] or exhibit a cytokine-like activity, e.g., vIL10 [29]. Interestingly, search for the most differentially expressed genes (data not shown) between EBV+ individuals and EBV-patients in identified MMP9 and IL18R1 in our study; these two genes have been shown to exhibit increased expression in sepsis survivors [30, 31], in relation to organ dysfunction [30] and inflammasome activation [31]. In contrast, EBV correlated with downregulation of IL-1β production in our patients; this mirrors BCG vaccination-induced trained immunity and the protective effect of IL-1β [18]. Last, there was an apparent association between EBV-TTV reactivation and the occurrence of HAI (46% of HAI versus 22% for non-EBV-TTV reactivation), which was not observed for EBV-HSV1 reactivation (29% of HAI versus 25% for non-EBV-HSV1 reactivation).

HSV1 showed a 25-fold and EBV a 4-fold cumulative incidence rate during the first week. In a previous study, the most rapid increase in detection rate (from virus negative to virus positive) of herpes viruses was observed for EBV [4]. CMV and HHV-6 had a slow rise in detection rates, as has been previously described [4]. Meanwhile, TTV only increased by 25% during the week; this contrasts with a 75% conversion rate at day 6 after sepsis observed in a previous study [4]. Nevertheless, the qualitative detection of viral DNAemia may be not sufficient to capture the whole picture, especially concerning TTV. For TTV viral loads greater than 10,000 cp/ml, 4 patients were EBV positive and 16 were EBV negative; this did not support the hypothesis that TTV replication may be stimulated by EBV [24]. In our study, among patients exhibiting these high TTV viral loads, 5 were non-survivors and 16 were survivors at D28; this differs from the observation that high TTV viral load is associated with increased 90-day mortality [4].

The current study is the first to describe precisely, with standardized procedures, the viremia from five herpes viruses and TTV in septic shock patients. As serology for CMV and HSV were not available in this cohort, we can only suggest that we observed viral reactivation. Systematic selection of patients with D1, D3, and D6 samples avoided any confounding due to early death during the first week in ICU and allowed tracking of viral DNAemia during this period. Conversely, as we included only patients still alive at day 5–7, we might have assessed a slightly less severe cohort of septic shock; hence, such a pilot cohort may not capture the diversity of septic shock patients. Of note, besides those parameters (ICU length of stay, survival D28, at least one IAI) presented in Additional file 1: Figure S1, there was no other differences in any clinical parameter collected at admission and presented elsewhere [16, 17]. Our results also underline that focusing only on the first week is not sufficient, as viral reactivation is probably a marker of persistent immune suppression. Although we developed a robust quantitative process to measure the viral load, mainly qualitative data was used to describe viral DNAemia. All herpes viruses were grouped to reflect the altered immune status, as they all share a viral cycle with latency and ability to reactivate. Nevertheless, such quantitative tools should allow the further definition of several thresholds to better discriminate between (1) non-significant viral load, (2) viral “reactivation” as a marker of immunosuppression, and (3) high viral loads supporting a true viral infection requiring treatment as previously discussed [32]. Validation of a similar approach during the whole ICU stay, and maybe after ICU release would provide insight into the expected dynamics of this viral reactivation. This can be achieved using a large unbiased cohort for which the immune status will be objectively defined, such as the REALISM project (NCT01931956). The REALISM project consists of a prospective longitudinal single-center clinical study which aims to provide an operational definition of injury-induced immunosuppression predicting clinically relevant outcomes [33].

## Conclusions

By applying a semi-automated process of viral DNAemia determination to this cohort of 98 septic shock patients, we observed that the number of patients with positive viremia increase during the first week in the ICU. Although there was no robust association with clinical outcomes, the presence of EBV not only appeared to be associated with HAI but also had an apparent protective effect against mortality. In comparison, viremia due to several herpes viruses simultaneously was associated with higher ICU mortality. This study, introducing standardized procedures from extraction to detection, provides the basis for future standardized diagnostic criteria. A prospective longitudinal single-center clinical study targeting a larger population size and a longer follow-up period will use these same procedures to robustly define for each virus thresholds to discriminate between non-significant viral load, viral reactivation, and high viral loads. This has the potential to provide an indication of the differing extent and duration of immunosuppression and/or treatment.

## Additional file

Additional file 1: **Table S1.** (A) Virus strain-reference panel and (B) viral PCR-reference panel used for determination of the LOD in the automated process. **Figure S1.** Selection of the so-called “viral cohort”, a subset of the MIP REA cohort. **Figure S2.** (A) Semi-automated procedure applied to detect DNA viremia in plasma. Internal controls are used all along the process to qualify each single step, namely extraction, amplification, detection. **Figure S3.** (A) Procedure applied to determine the LOD (B) Last performed dilution giving a positive count and selection (red) of the LOD (left), and amplification on blood extracts from healthy volunteers; ^a^ TTV LOD in plasma was derived from Kulifaj D. et al., 2018 (https://doi.org/10.1016/j.jcv.2018.06.010). **Table S2.** Association between binary endpoints or markers and viremia presence. Clinical outcomes are mortality at D28 following ICU admission and HAI occurrence occurring during the hospitalization. Biomarkers, quantified by RT-PCR, consisted of CD74 ratio of D3/D1 (> 1.238 = increased incidence of HAI), CX3CR1 measured at D3 (> 0.253 = increased incidence of mortality at D28), IL10 measured at D3 (> 0.039 = increased incidence of HAI), and IL1b measured at D3 (increase at D2–D4 = increase incidence of HAI in pediatric patients). Thresholds were determined using the total MIPREA cohort, for CD74 and IL10 [16] and CX3CR1 as well [17, 19]. As no threshold was proposed for IL1β [19], it was used as a continuous variable. ^a^IL1β not a binary endpoint. (PPT 1049 kb)

## Abbreviations

CCTIRS: Comité Consultatif sur le Traitement de l’Information en matière de Recherche en Santé
CMV: Cytomegalovirus
CNIL: Comission Nationale Informatique et Liberté
CT: Crossing threshold
DNA: Desoxyribonucleic acid
EBV: Ebstein-Barr virus
EFS: Etablissement Français du Sang
HAI: Healthcare-associated infection
HHV: Human herpes virus
HSV: Herpes simplex virus
IC: Internal control
ICU: Intensive care unit
LOD: Limit of detection
LOQ: Limit of quantification
PCR: Polymerase chain reaction
QS: Quantification standard
SC: Sensitivity control
SOFA: Sequential Organ Failure Assessment score
TTV: Torque teno virus
