# Supplementary material for: Early herpes and TTV DNAemia in septic shock patients: a pilot study
Source: Intensive Care Med Exp. 2019 May 18;7:28. doi: 10.1186/s40635-019-0256-z (PMC6525672; doi:10.1186/s40635-019-0256-z)
Supplement: Supplementary file 1 — Table S1. (A) Virus strain-reference panel and (B) viral PCR-reference panel used for determination of the LOD in the automated process. Figure S1. Selection of the so-called “viral cohort”, a subset of the MIP REA cohort. Figure S2. (A) Semi-automated procedure applied to detect DNA viremia in plasma. Internal controls are used all along the process to qualify each single step, namely extraction, amplification, detection. Figure S3. (A) Procedure applied to determine the LOD (B) Last performed dilution giving a positive count and selection (red) of the LOD (left), and amplification on blood extracts from healthy volunteers; a TTV LOD in plasma was derived from Kulifaj D. et al., 2018 (https://doi.org/10.1016/j.jcv.2018.06.010). Table S2. Association between binary endpoints or markers and viremia presence. Clinical outcomes are mortality at D28 following ICU admission and HAI occurrence occurring during the hospitalization. Biomarkers, quantified by RT-PCR, consisted of CD74 ratio of D3/D1 (> 1.238 = increased incidence of HAI), CX3CR1 measured at D3 (> 0.253 = increased incidence of mortality at D28), IL10 measured at D3 (> 0.039 = increased incidence of HAI), and IL1b measured at D3 (increase at D2–D4 = increase incidence of HAI in pediatric patients). Thresholds were determined using the total MIPREA cohort, for CD74 and IL10 [16] and CX3CR1 as well [17, 19]. As no threshold was proposed for IL1β [19], it was used as a continuous variable. aIL1β not a binary endpoint. (PPT 1049 kb) [file 40635_2019_256_MOESM1_ESM.ppt]

## Slide 1
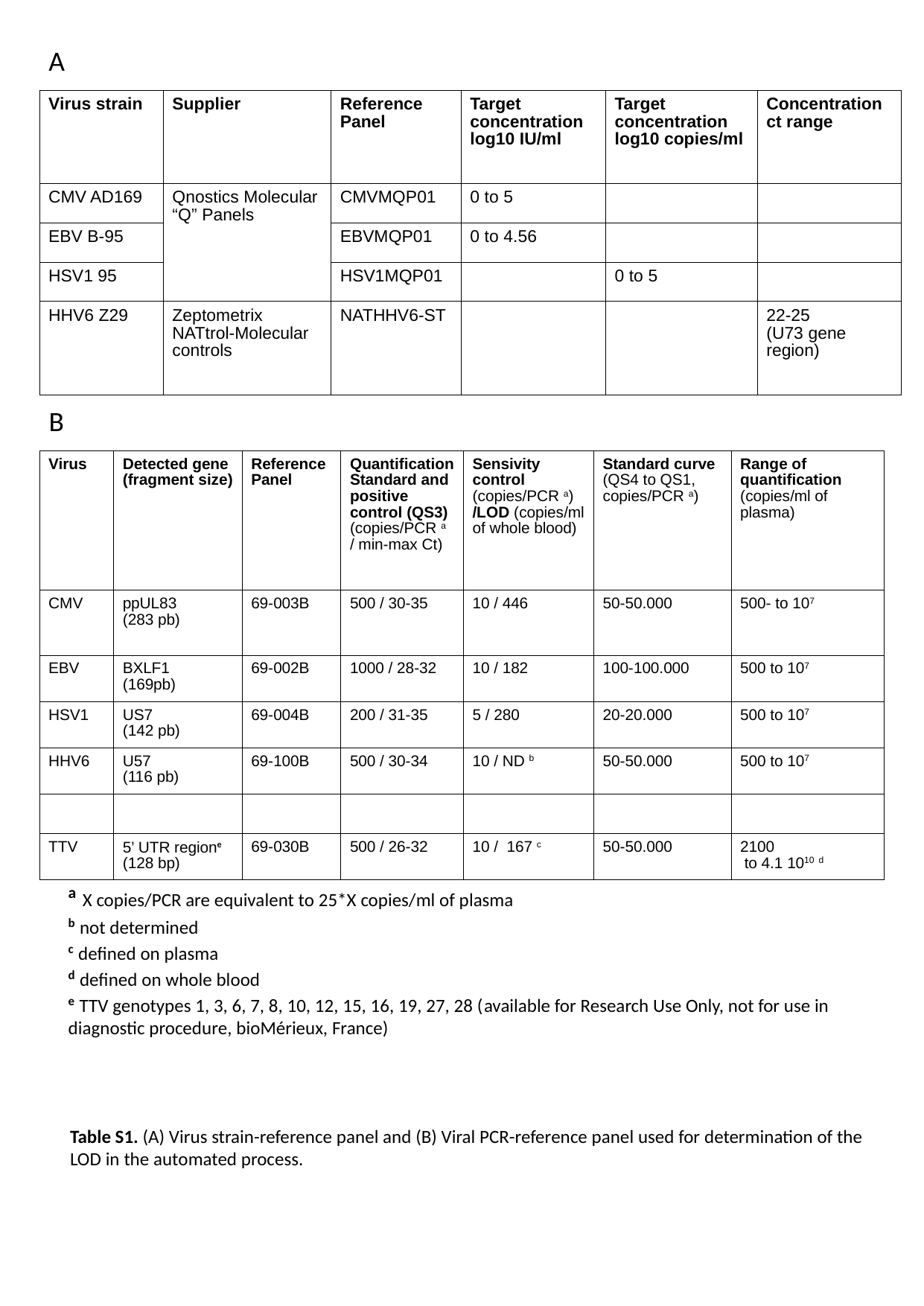

A
| Virus strain | Supplier | Reference Panel | Target concentration log10 IU/ml | Target concentration log10 copies/ml | Concentration ct range |
| --- | --- | --- | --- | --- | --- |
| CMV AD169 | Qnostics Molecular “Q” Panels | CMVMQP01 | 0 to 5 | | |
| EBV B-95 | | EBVMQP01 | 0 to 4.56 | | |
| HSV1 95 | | HSV1MQP01 | | 0 to 5 | |
| HHV6 Z29 | Zeptometrix NATtrol-Molecular controls | NATHHV6-ST | | | 22-25 (U73 gene region) |
B
| Virus | Detected gene (fragment size) | Reference Panel | Quantification Standard and positive control (QS3) (copies/PCR a / min-max Ct) | Sensivity control (copies/PCR a) /LOD (copies/ml of whole blood) | Standard curve (QS4 to QS1, copies/PCR a) | Range of quantification (copies/ml of plasma) |
| --- | --- | --- | --- | --- | --- | --- |
| CMV | ppUL83 (283 pb) | 69-003B | 500 / 30-35 | 10 / 446 | 50-50.000 | 500- to 107 |
| EBV | BXLF1 (169pb) | 69-002B | 1000 / 28-32 | 10 / 182 | 100-100.000 | 500 to 107 |
| HSV1 | US7 (142 pb) | 69-004B | 200 / 31-35 | 5 / 280 | 20-20.000 | 500 to 107 |
| HHV6 | U57 (116 pb) | 69-100B | 500 / 30-34 | 10 / ND b | 50-50.000 | 500 to 107 |
| | | | | | | |
| TTV | 5’ UTR regione (128 bp) | 69-030B | 500 / 26-32 | 10 / 167 c | 50-50.000 | 2100 to 4.1 1010 d |
a X copies/PCR are equivalent to 25*X copies/ml of plasma
b not determined
c defined on plasma
d defined on whole blood
e TTV genotypes 1, 3, 6, 7, 8, 10, 12, 15, 16, 19, 27, 28 (available for Research Use Only, not for use in diagnostic procedure, bioMérieux, France)
Table S1. (A) Virus strain-reference panel and (B) Viral PCR-reference panel used for determination of the LOD in the automated process.

## Slide 2
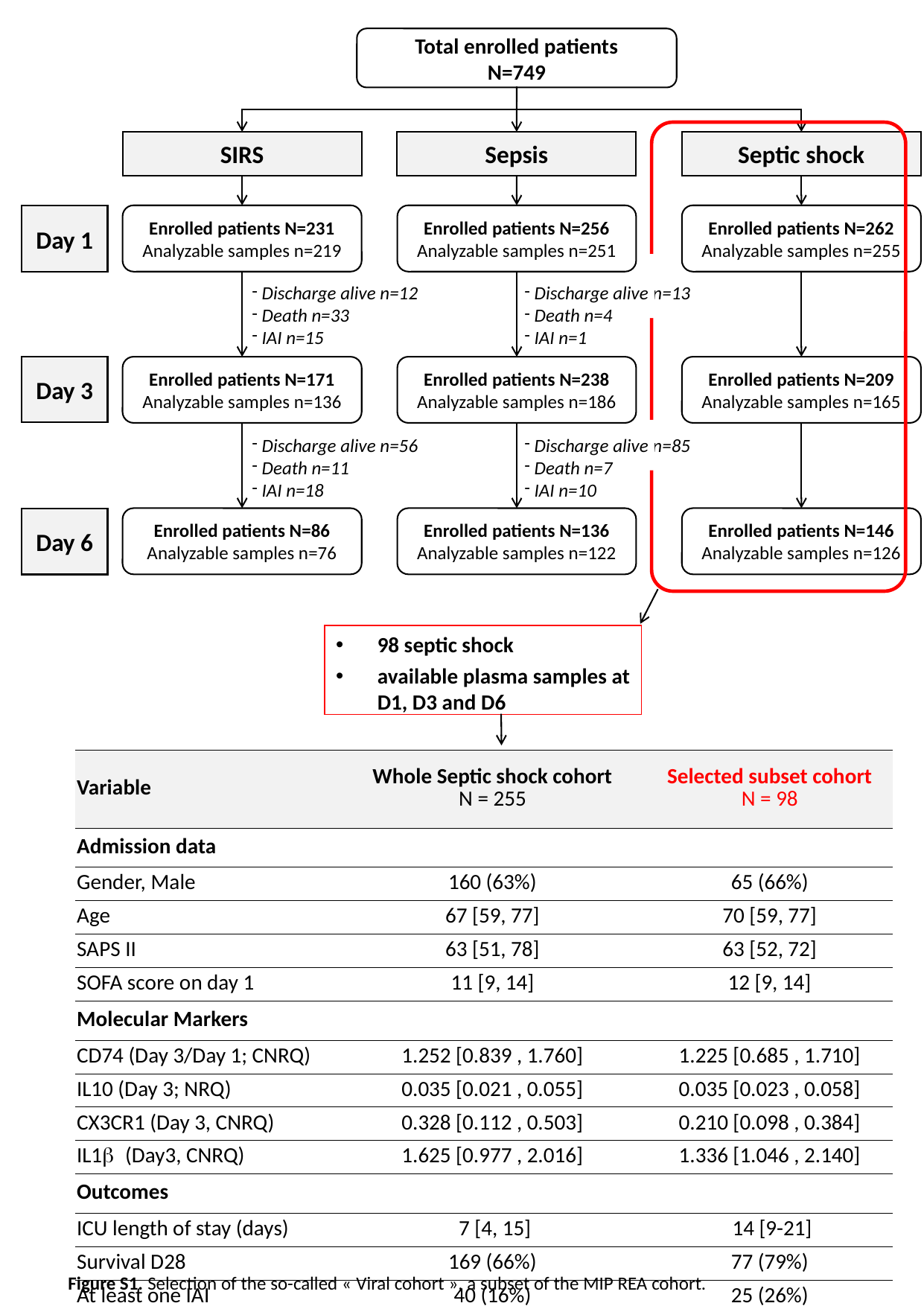

Total enrolled patients
N=749
SIRS
Sepsis
Septic shock
Enrolled patients N=231
Analyzable samples n=219
Enrolled patients N=256
Analyzable samples n=251
Enrolled patients N=262
Analyzable samples n=255
Day 1
Discharge alive n=12
Death n=33
IAI n=15
Discharge alive n=13
Death n=4
IAI n=1
Day 3
Enrolled patients N=171
Analyzable samples n=136
Enrolled patients N=238
Analyzable samples n=186
Enrolled patients N=209
Analyzable samples n=165
Discharge alive n=56
Death n=11
IAI n=18
Discharge alive n=85
Death n=7
IAI n=10
Enrolled patients N=86
Analyzable samples n=76
Enrolled patients N=136
Analyzable samples n=122
Enrolled patients N=146
Analyzable samples n=126
Day 6
98 septic shock
available plasma samples at D1, D3 and D6
| Variable | Whole Septic shock cohort N = 255 | Selected subset cohort N = 98 |
| --- | --- | --- |
| Admission data | | |
| Gender, Male | 160 (63%) | 65 (66%) |
| Age | 67 [59, 77] | 70 [59, 77] |
| SAPS II | 63 [51, 78] | 63 [52, 72] |
| SOFA score on day 1 | 11 [9, 14] | 12 [9, 14] |
| Molecular Markers | | |
| CD74 (Day 3/Day 1; CNRQ) | 1.252 [0.839 , 1.760] | 1.225 [0.685 , 1.710] |
| IL10 (Day 3; NRQ) | 0.035 [0.021 , 0.055] | 0.035 [0.023 , 0.058] |
| CX3CR1 (Day 3, CNRQ) | 0.328 [0.112 , 0.503] | 0.210 [0.098 , 0.384] |
| IL1β (Day3, CNRQ) | 1.625 [0.977 , 2.016] | 1.336 [1.046 , 2.140] |
| Outcomes | | |
| ICU length of stay (days) | 7 [4, 15] | 14 [9-21] |
| Survival D28 | 169 (66%) | 77 (79%) |
| At least one IAI | 40 (16%) | 25 (26%) |
Figure S1. Selection of the so-called « Viral cohort », a subset of the MIP REA cohort.

## Slide 3
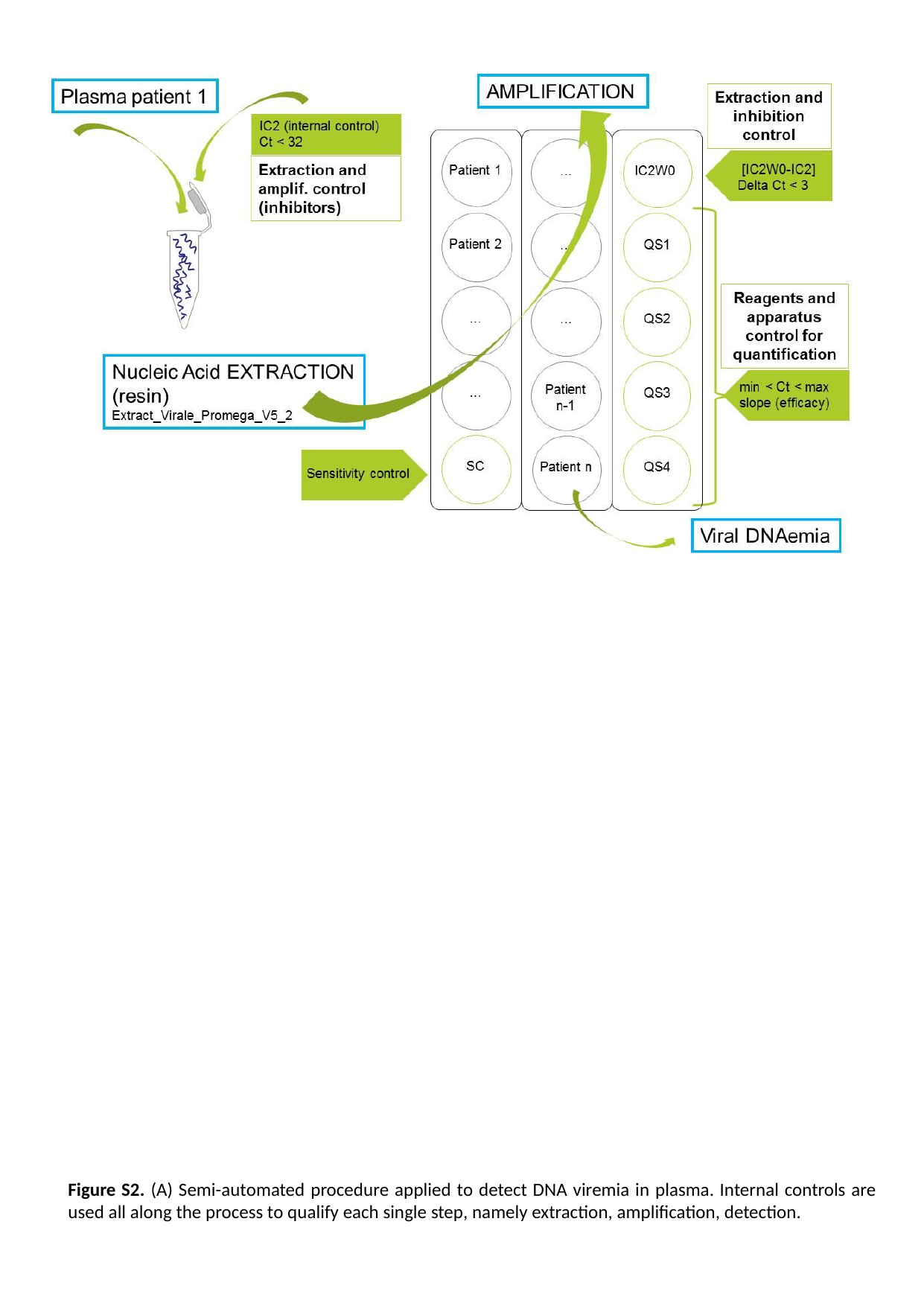

Figure S2. (A) Semi-automated procedure applied to detect DNA viremia in plasma. Internal controls are used all along the process to qualify each single step, namely extraction, amplification, detection.

## Slide 4
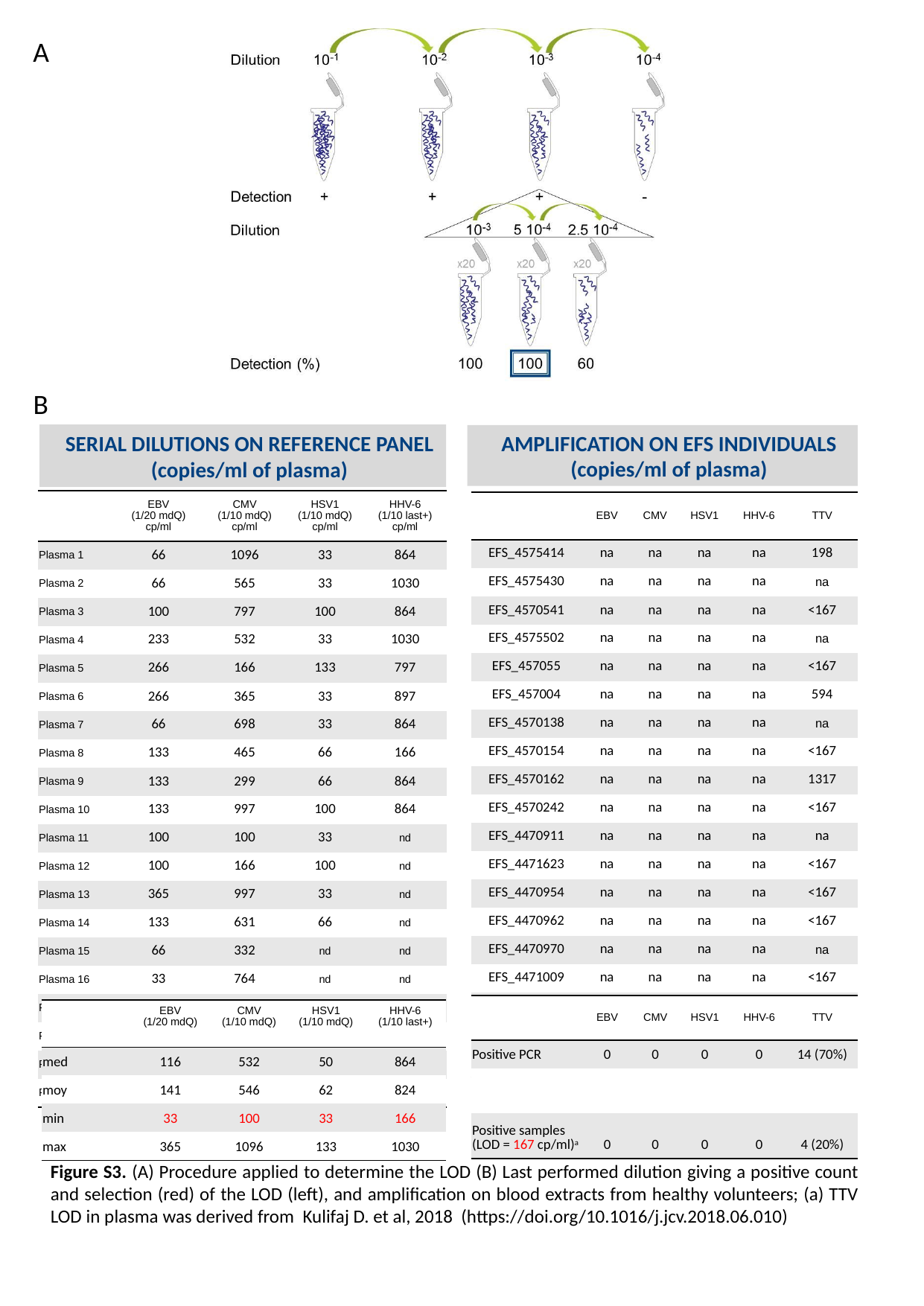

A
B
SERIAL DILUTIONS ON REFERENCE PANEL
(copies/ml of plasma)
AMPLIFICATION ON EFS INDIVIDUALS (copies/ml of plasma)
| | EBV (1/20 mdQ) cp/ml | CMV (1/10 mdQ) cp/ml | HSV1 (1/10 mdQ) cp/ml | HHV-6 (1/10 last+) cp/ml |
| --- | --- | --- | --- | --- |
| Plasma 1 | 66 | 1096 | 33 | 864 |
| Plasma 2 | 66 | 565 | 33 | 1030 |
| Plasma 3 | 100 | 797 | 100 | 864 |
| Plasma 4 | 233 | 532 | 33 | 1030 |
| Plasma 5 | 266 | 166 | 133 | 797 |
| Plasma 6 | 266 | 365 | 33 | 897 |
| Plasma 7 | 66 | 698 | 33 | 864 |
| Plasma 8 | 133 | 465 | 66 | 166 |
| Plasma 9 | 133 | 299 | 66 | 864 |
| Plasma 10 | 133 | 997 | 100 | 864 |
| Plasma 11 | 100 | 100 | 33 | nd |
| Plasma 12 | 100 | 166 | 100 | nd |
| Plasma 13 | 365 | 997 | 33 | nd |
| Plasma 14 | 133 | 631 | 66 | nd |
| Plasma 15 | 66 | 332 | nd | nd |
| Plasma 16 | 33 | 764 | nd | nd |
| Plasma 17 | 199 | 532 | nd | nd |
| Plasma 18 | 66 | 399 | nd | nd |
| Plasma 19 | 233 | 731 | nd | nd |
| Plasma 20 | 66 | 299 | nd | nd |
| | EBV | CMV | HSV1 | HHV-6 | TTV |
| --- | --- | --- | --- | --- | --- |
| EFS\_4575414 | na | na | na | na | 198 |
| EFS\_4575430 | na | na | na | na | na |
| EFS\_4570541 | na | na | na | na | <167 |
| EFS\_4575502 | na | na | na | na | na |
| EFS\_457055 | na | na | na | na | <167 |
| EFS\_457004 | na | na | na | na | 594 |
| EFS\_4570138 | na | na | na | na | na |
| EFS\_4570154 | na | na | na | na | <167 |
| EFS\_4570162 | na | na | na | na | 1317 |
| EFS\_4570242 | na | na | na | na | <167 |
| EFS\_4470911 | na | na | na | na | na |
| EFS\_4471623 | na | na | na | na | <167 |
| EFS\_4470954 | na | na | na | na | <167 |
| EFS\_4470962 | na | na | na | na | <167 |
| EFS\_4470970 | na | na | na | na | na |
| EFS\_4471009 | na | na | na | na | <167 |
| EFS\_4472087 | na | na | na | na | 216 |
| EFS\_4472108 | na | na | na | na | <167 |
| EFS\_4472116 | na | na | na | na | <167 |
| | EBV | CMV | HSV1 | HHV-6 | TTV |
| --- | --- | --- | --- | --- | --- |
| Positive PCR | 0 | 0 | 0 | 0 | 14 (70%) |
| | | | | | |
| Positive samples (LOD = 167 cp/ml)a | 0 | 0 | 0 | 0 | 4 (20%) |
| | EBV (1/20 mdQ) | CMV (1/10 mdQ) | HSV1 (1/10 mdQ) | HHV-6 (1/10 last+) |
| --- | --- | --- | --- | --- |
| med | 116 | 532 | 50 | 864 |
| moy | 141 | 546 | 62 | 824 |
| min | 33 | 100 | 33 | 166 |
| max | 365 | 1096 | 133 | 1030 |
Figure S3. (A) Procedure applied to determine the LOD (B) Last performed dilution giving a positive count and selection (red) of the LOD (left), and amplification on blood extracts from healthy volunteers; (a) TTV LOD in plasma was derived from Kulifaj D. et al, 2018 (https://doi.org/10.1016/j.jcv.2018.06.010)

## Slide 5
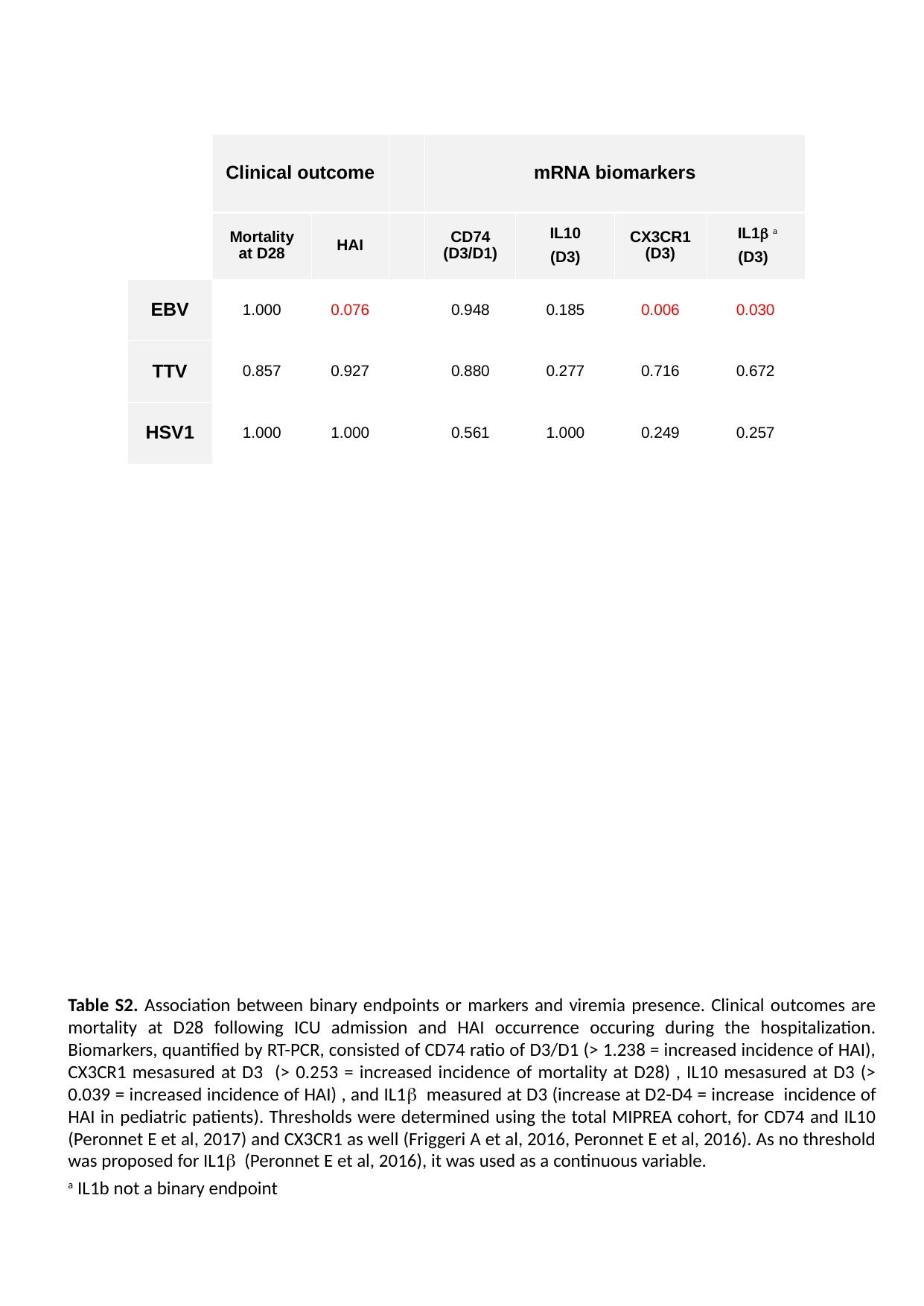

| | Clinical outcome | | | mRNA biomarkers | | | |
| --- | --- | --- | --- | --- | --- | --- | --- |
| | Mortality at D28 | HAI | | CD74 (D3/D1) | IL10 (D3) | CX3CR1 (D3) | IL1β a (D3) |
| EBV | 1.000 | 0.076 | | 0.948 | 0.185 | 0.006 | 0.030 |
| TTV | 0.857 | 0.927 | | 0.880 | 0.277 | 0.716 | 0.672 |
| HSV1 | 1.000 | 1.000 | | 0.561 | 1.000 | 0.249 | 0.257 |
Table S2. Association between binary endpoints or markers and viremia presence. Clinical outcomes are mortality at D28 following ICU admission and HAI occurrence occuring during the hospitalization. Biomarkers, quantified by RT-PCR, consisted of CD74 ratio of D3/D1 (> 1.238 = increased incidence of HAI), CX3CR1 mesasured at D3 (> 0.253 = increased incidence of mortality at D28) , IL10 mesasured at D3 (> 0.039 = increased incidence of HAI) , and IL1β measured at D3 (increase at D2-D4 = increase incidence of HAI in pediatric patients). Thresholds were determined using the total MIPREA cohort, for CD74 and IL10 (Peronnet E et al, 2017) and CX3CR1 as well (Friggeri A et al, 2016, Peronnet E et al, 2016). As no threshold was proposed for IL1β (Peronnet E et al, 2016), it was used as a continuous variable.
a IL1b not a binary endpoint
